# Effect of Sr on Microstructure and Strengthening Mechanism of Al-4.6Mg Alloy

**DOI:** 10.3390/ma16155450

**Published:** 2023-08-03

**Authors:** Zhanshou Yang, Yaping Dong, Wu Li, Xin Liu, Haitao Feng

**Affiliations:** 1 Key Laboratory of Comprehensive and Highly Efficient Utilization of Salt Lake Resources, Qinghai Institute of Salt Lakes, Chinese Academy of Sciences, Xining 810008, China; 2 Qinghai Engineering and Technology Research Center of Comprehensive Utilization of Salt Lake Resources, Xining 810008, China

**Keywords:** Al-4.6Mg alloy, Sr, microstructure, mechanical properties, strengthening mechanism

## Abstract

The as-cast Al-4.6Mg alloy was subjected to deformation and sensitization–desensitization heat treatment, and then the microstructure and the enhancement mechanism of Sr were investigated by optical microscopy, scanning electron microscopy–energy-dispersive spectroscopy, electron backscatter diffraction, and transmission electron microscopy. The precipitation phases of Al-4.6Mg alloy were mainly β-Al_3_Mg_2_, Al_6_Mn, and Al_6_(Mn Cr), and the nanoscale precipitation phases were Al_3_Mn and Al_11_Mn_4_. The formation of β-Al_3_Mg_2_ was hindered by the addition of 0.1 wt.% Sr. In addition, the precipitate phase Al_4_Sr and the nano-sized precipitate phase τ-Al_38_Mg_58_Sr_4_ were uniformly distributed in the spherical matrix. The addition of Sr promoted the redissolution of Mg atoms in Al-4.6Mg alloy, increasing the solubility of Mg in the α-Al matrix from 4.7 wt.% to 5.1 wt.%. The microstructure analysis showed that Sr addition inhibited the recovery and recrystallization of the alloy because the Sr element elevated the recrystallization temperature. As a result, the grain deformation was intensified, the grain size was decreased from 6.96 μm to 5.39 μm, the low-angle grain boundaries were increased from 78.7 at % to 84.6 at %, and the high-angle grain boundaries were increased from 21.3 at % to 15.4 at %. Furthermore, the mechanical properties of the alloy were significantly improved, and the plasticity degraded after the addition of the Sr element. The yield strength of the alloy was enhanced mainly through fine grain strengthening, dispersion strengthening, solid solution strengthening, and working hardening. The strengthening mechanisms were analyzed in detail.

## 1. Introduction

Al-Mg alloy has excellent specific strength, specific stiffness, weldability, intergranular corrosion (IGC) resistance, and stress corrosion resistance [1,2]. It also demonstrates great machinability and high fatigue strength. With these properties, Al-Mg alloy is extensively used in aviation, shipping, construction, transportation, and rail transit [3,4,5]. However, Mg is supersaturated in the matrix of Al-Mg alloy (e.g., Al-4.6Mg alloy). The excess Mg diffuses to form the β-Al_3_Mg_2_ phase when exposed to high temperatures (50–200 °C) for a long time [6]. The continuous grain boundary distribution of β-Al_3_Mg_2_ makes Al-Mg alloy prone to IGC and stress corrosion [7]. Because the β-Al_3_Mg_2_ phase is incoherent with the matrix, the Al-Mg alloy cannot be strengthened by heat treatment but by solid solution and work hardening [8]. The density of β-Al_3_Mg_2_ intermetallics is low (2.2 g/cm^3^), and the coarse β-Al_3_Mg_2_ is unfavorable for the improvement of the alloy’s mechanical properties [9]. For Al-Mg alloys, the mechanical properties and corrosion resistance can be improved by different thermomechanical treatments, local reversion of thermal treatments, and microalloying [8,10,11]. 

As a microalloying element, Sr is widely used in Al-Si alloys to modify their microstructures and improve their thermal conductivity and mechanical properties [12]. The effect of Sr on Al-Mg alloy was first reported in 2014. The addition of Sr significantly reduced the anodic kinetics of the Al-4Mg-0.4Mn alloy and improved its IGC resistance [13,14]. In the presence of Sr, Al_19_Mg_29_Sr_2_ phases were formed, reducing the β-Al_3_Mg_2_ phases and suppressing their continuous distribution at grain boundaries. Gupta, R.K. [15] investigated the effect of Sr content on the sensitizing behavior and corrosion resistance of Al-4.4Mg-0.5Mn alloy by theoretical calculations and practical experiments. The results showed that the addition of Sr caused the formation of the τ-Al_38_Mg_58_Sr_4_ phase during the sensitizing treatment and the consumption of Mg, leading to a decrease in the precipitated β-Al_3_Mg_2_ phase. The τ-Al_38_Mg_58_Sr_4_ phase and Al_19_Mg_29_Sr_2_ phase are identical in Al-Mg alloy. Che [8] explored the effect of Sr addition on the microstructure and mechanical properties of Al-2.4Mg alloy during hot extrusion and found that the grain size could be refined and homogenized by adding Sr. Under isothermal compression, Sr significantly affects the microstructure and texture of Al-4.6Mg alloy. The main textures of Al-4.6Mg alloy are the Goss texture and cubic texture, and the cubic texture and brass texture would be replaced after adding Sr [16].

The size, shape, and distribution of the second-phase particles are important for the mechanical properties of Al-Mg alloys. The second phase affects the recrystallization during particle-stimulated nucleation, further complicating the microstructure evolution and performance alteration of the alloys [17]. The modification effect of Sr and the strengthening effect of fine grains may be limited since Al-Mg alloys do not contain Sr. The corrosion resistance of Al-Mg alloys has been extensively studied, while the effect of Sr on the microstructural evolution of Al-Mg alloys and the influence of the size, shape, and distribution of the second-phase particles have received less attention [2]. In the present study, the effects of Sr on the microstructure, second-phase morphology, and mechanical properties of Al-4.6Mg alloy were studied under sensitized-desensitized heat treatment. The strengthening mechanisms of Sr on Al-4.6Mg alloy were discussed, including fine grain strengthening, dispersion strengthening, solid solution strengthening, and working hardening. This study provides a theoretical basis for the application of Sr in the industrial production of Al-Mg alloy.

## 2. Experimental Procedure

### 2.1. Alloy Preparation and Elemental Analysis

We report the melting method and dimensions of the as-cast Al-4.6Mg alloy. Its composition was analyzed by X-ray fluorescence spectrometry [2]. The main composition of Al-4.6Mg alloy is comparable to that of Al-4.6Mg-0.1Sr alloy, which contains Sr elements. The chemical composition of Al-4.6Mg-0.1Sr alloy is shown in Table 1.

### 2.2. Alloy Rolling and Annealing Heat Treatment

After homogenization heat treatment (510 °C/7.5 h), ingots with dimensions of 240 mm × 180 mm × 45 mm were hot-rolled at 480 °C. Their thickness was reduced to 9 mm in 10 passes, and the hot rolling deformation was 77.7%. Then, they were held in a furnace at 460 °C for 30 min, naturally cooled to 100 °C, and then cold-rolled to a thickness of 4 mm, with cold rolling deformation of 55.6%. The annealing heat treatment was carried out in two steps:

(1) Sensitization treatment at 100 °C for 100 h;
(2) Desensitization at 250 °C for 1 h.

The two-step process is referred to as sensitization–desensitization heat treatment.

### 2.3. Sample Analysis

The rolled alloy material was cut into cubes of 10 mm × 8 mm × 4 mm by electrical discharge machining, cleaned with acetone for 10 min, and then subjected to sensitization–desensitization heat treatment. The samples were first polished with 400# sandpaper to remove the oxide layer, and then they were subjected to further polishing using different grades of sandpaper; the polishing continued until the samples were polished with 5000# sandpaper. Then, the alloy material was polished with 0.05-μm polishing solution for 3 min and electrolytically polished at −30 °C for 120 s (electrolyte: 17% perchloric acid + 83% methanol). The microstructure evolution after rolling deformation was analyzed by electron backscatter diffractometry (EBSD) (Oxford-ebsd, Oxford Instruments, Abingdon, UK) and low-vacuum scanning electron microscopy (SEM) (JSM-5610LV/INCA, Oxford Instruments, Abingdon, UK)–energy-dispersive spectroscopy (EDS) (JSM-5610LV/INCA, Oxford Instruments, Abingdon, UK) (SEM-EDS); alloy specimens were cut by a focused ion beam, and their dislocations and precipitated phases were explored by transmission electron microscopy (TEM). (JEM-F200, JEOL, Tokyo, Japan).

### 2.4. Alloy Material Mechanical Property Test

The mechanical properties of the alloy specimens were tested according to the China national standard GB228.1-2002 after deformation and sensitization–desensitization heat treatment, at a tensile rate of 2 mm/min, along the rolling direction. The average value of three specimens in the same batch was taken as the test result.

## 3. Results and Discussion

### 3.1. Microstructure Analysis

**Figure 1:**
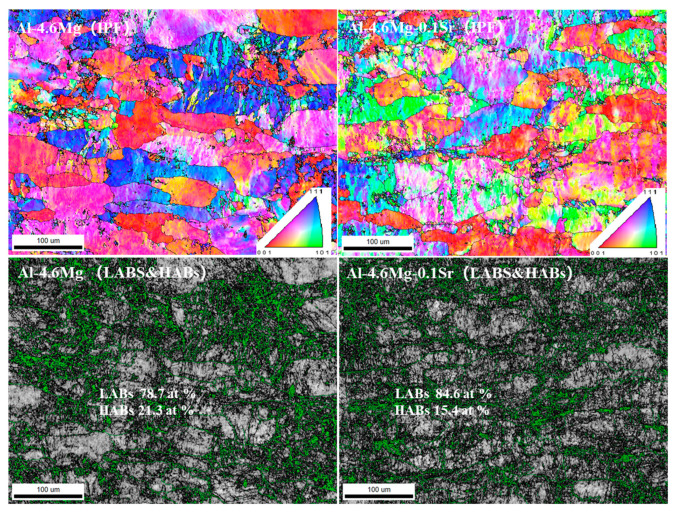
IPF and LAB and HAB images of the two alloys.

Figure 1 shows a diagram of the inverse pole figure (IPF) and images of the low-angle grain boundaries (LABs) and high-angle grain boundaries (HABs) of the two alloys in the sensitized–desensitized heat treatments. The IPF diagram reveals a distinct grain orientation, clear grain boundary, and some anisotropy in the grain orientation. The microstructure of the alloy is mainly composed of equiaxed grains, and the fiber structure of the rolled grains is gradually diluted, but deformed grains exist along the rolling direction. The Al-4.6Mg alloy has higher content of grains in the <001> orientation and the <111> orientation, but lower content in the <101> orientation. Due to the addition of Sr elements, significant transformation occurs, with a substantial grain decrease in the <111> orientation, a slight decrease in the <001> orientation, and a remarkable increase in the <101> orientation. The significant increase in the <101> orientation is attributed to the adsorption of Sr on the surface.

The grain boundary distribution of the alloys under sensitization–desensitization heat treatment is shown in Figure 1. The black and green lines represent LABs and HABs, respectively; the orientation angle of LABs ranges from 2 to 15° and that of HABs is greater than 15°. The Al-4.6Mg alloy had LABs of 78.7 wt.% and HABs of 21.3 wt.%; the Al-4.6Mg-0.1Sr alloy had LABs of 84.6 wt.% and HABs of 15.4 wt.%. The results indicate that adding Sr to Al-4.6Mg alloy increases the LABs and decreases the HABs. The enrichment of LABs within the grains suggests the presence of many deformed grains within the material, which is possibly due to continuous rolling deformation. This change enables LABs in Al-Mg alloys to spread from the boundary to the grain center [18]. The increase in LABs indicates a gradual elevation in defect density within the material, demonstrating that the presence of deformed tissue and a strong texture increases the ability of the dislocations to slip. Al-4.6Mg alloys have more HABs, associated with the Mg-rich phase (β-Al_3_Mg_2_). The in-situ heating TEM experiments showed that the LABs of Al-Mg alloys can potentially diffuse from the boundary to the grain center [18]. The Mg-rich phase has a faster growth rate and a larger size at HABs than at LABs, resulting in an increased number of HABs [19]. As a result, HABs have a larger size and more Mg-rich phases. Near the HAB-enriched grain boundaries, the deformed and subgranular grains were transformed into HABs through dynamic recovery and dynamic recrystallization. This result indicates that the material underwent recovery and recrystallization after sensitized–desensitized treatment, leading to a decreased anti-dislocation ability, increased internal stress, and enhanced stability.

**Figure 2:**
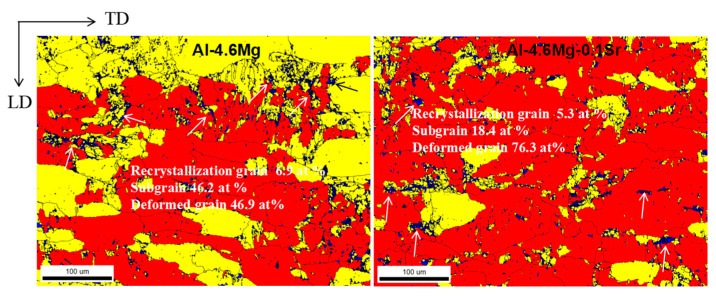
The grain orientation spread images of the two alloys.

The grain orientation spread method was adopted to analyze the dynamic recrystallization behavior of the alloy materials (Figure 2). Essentially, the discrepancy between the average grain orientation and all measurements was determined [20]. This method can also distinguish recrystallized grains from deformed grains and determine their volume fractions [21]. The blue grains represent recrystallization grains when the orientation difference angle is between 0° and 2° (marked by arrows); the yellow grains represent subgrains when the orientation angle is between 2° and 7°; the red grains represent deformed grains when the orientation angle is larger than 7°. The recrystallization grains of Al-4.6Mg alloy account for 6.9 wt.%, the subgrains account for 46.2 wt.%, and the deformed grains account for 46.9%. In the Al-4.6Mg-0.1Sr alloy, the recrystallization grains account for 5.3 wt.%, the subgrains comprise 18.4 wt.%, and the deformed grains constitute 76.3 wt.%. After deformation, the Al-4.6Mg alloy had a higher degree of dynamic recovery (DRV) and dynamic recrystallization (DRX) under the sensitization–desensitization heat treatment, and the volume fraction of recrystallized grains and substructure grains increased. The Al-4.6Mg-0.1Sr alloy had a large number of dislocations generated during the deformation process, and a high stacking fault energy was required to complete DRV and DRX due to the addition of the Sr element, which increased the recrystallization temperature of the alloy, and there existed a large number of deformed grains generated during rolling. The average grain size of the two alloys was analyzed by the TSL OIM software. The average grain size of Al-4.6Mg alloy was 6.86 μm, while that of Al-4.6Mg-0.1Sr alloy was 5.39 μm, indicating that the Sr element increased the recovery temperature and recrystallization temperature, restrained the recovery and recrystallization, increased the deformed grains, and refined the grains.

**Figure 3:**
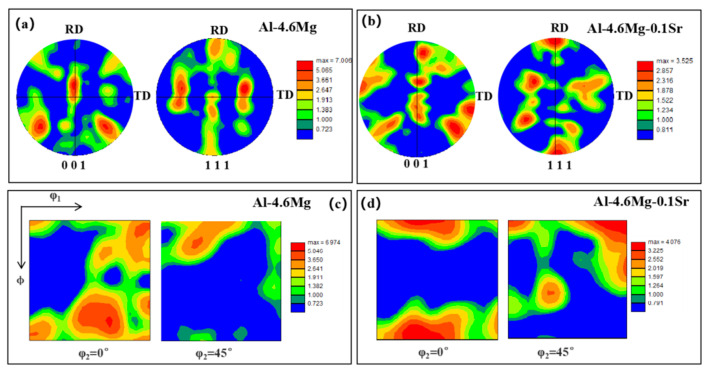
The pole images and ODF images of the two alloys. (**a**) The pole image of Al-4.6Mg alloy, (**b**) The pole image of Al-4.6Mg alloy, (**c**) The ODF image of Al-4.6Mg alloy, (**d**) The ODF image of Al-4.6Mg-0.1Sr alloy.

The {001} and {111} polar diagrams and the orientation distribution function (ODF) diagrams at φ2 = 0° and φ2 = 45° are plotted in Figure 3. By comparing the plotted polar diagrams with the standard ones [22,23], it is found that the textures of Al-4.6Mg alloy are concentrated in the directions of (001), (111), and (110), and the S{123}<634> texture emerges (Figure 3a,b) under the sensitization–desensitization heat treatment. The cubic grain with the S{123}<634> texture presents a stable orientation after deformation rotation, which can coordinate or maintain the stability of orthogonality. It may also be a potential nucleation site for recrystallization. The relationship between S{123}<634> and grains is approximately 40°<111>, contributing to good formability. The texture of the Al-4.6Mg-0.1Sr alloy is concentrated in the (110) direction and (001) direction, which is parallel to the TD direction. The results also illustrate that the (111) texture of the alloy decreases with the addition of the Sr element, indicating the inhibitory effect on the (111) texture during recrystallization.

Figure 3c,d show the ODF images of the alloys at φ2 = 0 ° and φ2 = 45 ° under sensitization–desensitization heat treatment. It can be observed from Figure 3c that the main textures of the Al-4.6Mg alloy consist of Cube{100}<001> (0.6 vol.%), r-Cube{001}<110> (3.2 vol.%), {258}<1$\overset{-}{2}$1> (0.001 vol.%), and {025}<100> (0.4 vol.%). In contrast, the textures of Al-4.6Mg-0.1Sr alloy mainly include Cube{100}<001> (1.4 vol.%), r-cube{001}<110> (3.9 vol.%), {258}<{1$\overset{-}{2}$1}> (1.4 vol.%), and {025}<100> (0.4 vol.%) (Figure 3d). The content of Cube{100}<001>, r-Cube{001}<110>, and {258}<1$\overset{-}{2}$1> increases with the addition of Sr. In the presence of Sr, the difference in stored energy in the process of stimulated nucleation enlarges, and nucleation occurs in the deformation region owing to the increase in Cube{100}<001>. Furthermore, Cube{100}<001> has lower storage energy [24] and 40°<111> favors boundary growth [23]. r-Cube{001}<110> is obtained by the rotation of the grain along the rolling direction by 45°, and this texture can also be achieved during cold deformation with a smaller Taylor factor [25]. We found that the addition of the Sr element increased the r-cube{001}<110> of Al-4.6Mg from 3.2 vol.% to 6.9 vol.% under annealing heat treatment (250°C/1h) [2], indicating that the Sr element has a significant effect on the r-cube{001}<110> of Al-4.6Mg alloy under annealing treatment.

### 3.2. Precipitate Phase Analysis

**Figure 4:**
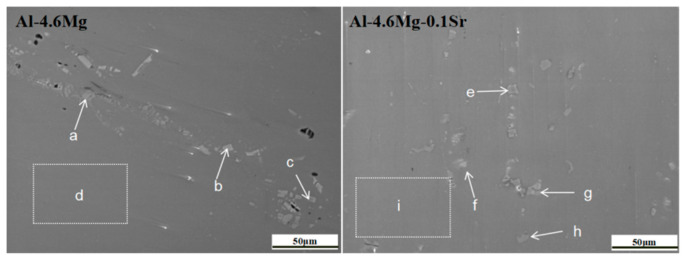
SEM-EDS analysis of the precipitated phases of the two alloys. (a–i corresponds to a–i in Table 2).

Figure 4 shows the SEM-EDS diagrams of the two alloys under the sensitized–desensitized heat treatment. The surface of the Al-4.6Mg alloy shows significant processing defects, voids, and a discontinuous precipitated phase distribution. The precipitated phase mainly comprises short rods, bars, blocks, and small discontinuous particles. The content of the second-phase elements was analyzed by EDS, and the results are shown in Table 2, where the letters correspond to different precipitated phases in Figure 4. The content of Al, Mg, and Mn in the block phase was 81.3 wt.%, 3.2 wt.%, and 13.6 wt.% at position a, respectively; the values were 79.8 wt.%, 2.5 wt.%, and 15.6 wt.% at position c, respectively. The results show that the block phases at positions a and c were the same, mainly including the β-Al_3_Mg_2_ and Al_6_(Mn Cr) phases. The β-Al_3_Mg_2_ intermetallic compound in Al-4.6Mg alloys has a low density and exhibits a high yield strength of 780 MPa at 225°C [26]. Changing the morphology and size of β-Al_3_Mg_2_ is the key to improving the mechanical properties and corrosion resistance of Al-4.6Mg alloy. Al, Mn, and Cr are the main elements of the block phase at position b. The high content of Mn and Cr indicates that the main phase is Al_6_(Mn Cr). The solubility of alloy elements in the α-Al matrix was obtained at position d, and the solid solubility of Mg and Mn was 4.7 wt.% and 0.6 wt.%, respectively.

The second phase of Al-4.6Mg-0.1Sr alloy was more uniformly distributed, and the defects on the alloy surface were reduced (Figure 4). This phase was mainly composed of short bars, spheres, and square phases. The content of Al, Mn, and Mg in the square phase was 80.9 wt.%, 15.7 wt.%, and 1.1 wt.% at position e, respectively; the values were 82.1 wt.%, 13.5 wt.%, and 2.2 wt.% at position g, respectively. The results show that the block-like phases at positions e and g are identical, mainly including β-Al_3_Mg_2_ and Al_6_Mn. The short rod phase at position f is mainly composed of Al (78.2 wt.%), Sr (13.3 wt.%), and Mg (5.2 wt.%), suggesting that the main component is the Al_4_Sr phase and a spherical nano-sized τ-Al_38_Mg_58_Sr_4_ phase. The main elements of the short rod phase at position h are Al (71.4 wt.%), Sr (4.4 wt.%), Mg (9.8 wt.%), and Mn (11.4 wt.%), indicating that the main phases are Al_4_Sr, Al_6_(Mn Cr) and τ-Al_38_Mg_58_Sr_4_. After adding 0.1 wt.% Sr to the Al-4.6Mg alloy, the solubility of Mg increased from 4.7 wt.% to 5.1 wt.%, and that of Cr decreased from 0.3 wt.% to 0.1 wt.%; there were no significant changes in the solubility of Mn (Table 2). These results indicate that the Sr element promotes the redissolution of the Mg-rich phase and the precipitation of the Cr-rich phase. The Al_4_Sr phase and spherical nano-sized τ-Al_38_Mg_58_Sr_4_ phase were precipitated from the α-Al matrix of the Al-4.6Mg-0.1Sr alloy. The τ-Al_38_Mg_58_Sr_4_ phase was formed by combining the Mg atom with Al_4_Sr by peritectic reaction—specifically, Mg(L) + Al_4_Sr = τ. This formation occurs at 460–473 °C and reduces the content of the Al_4_Sr phase from 42% to 35% [16,27]. The new phase precipitates at grain boundaries and inside grains, hindering the dislocation movement and increasing the shear stress required by the dislocation.

### 3.3. TEM Analysis of Precipitates

**Figure 5:**
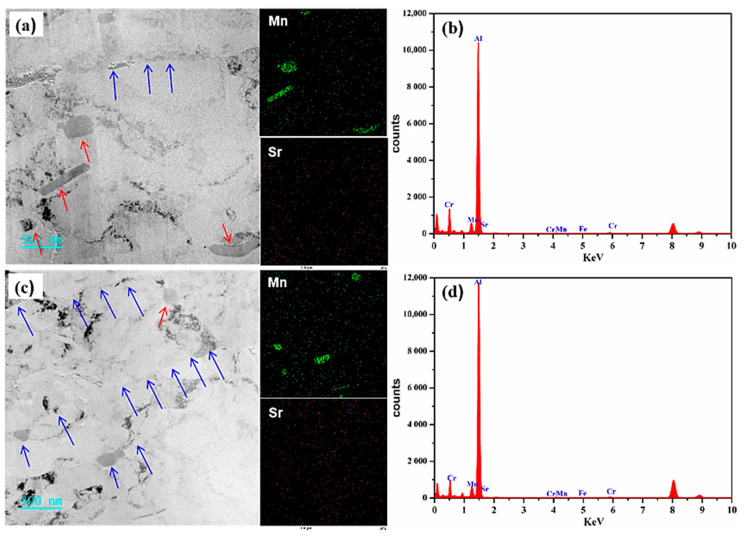
TEM-EDS analysis of Al-4.6Mg alloy. (**a**,**b**) TEM and Mapping images of Al-4.6Mg alloy, (**c**,**d**) EDS images of Al-4.6Mg alloy.

To further explore the nano-precipitates of Al-4.6Mg alloy in sensitized–desensitized heat treatment, TEM-EDS analysis was performed on the two alloys. Figure 5 shows the TEM-EDS diagram of Al-4.6Mg alloy. The precipitates of Al-4.6Mg alloy are mainly composed of bars, blocks, spheres, and square phases. The grain boundary and phase boundary (marked by white arrows) can be observed in the TEM bright-field phase diagram. From the TEM-EDS image, it can be found that the precipitated phases are mainly Mn-rich and Mg-rich phases, marked by yellow and red arrows in Figure 5a,c, respectively. The distributions of these phases are clearly displayed in the diagram with different distribution patterns, indicating the differences in the nucleation mode, growth direction, and element content of the precipitated phases. The corresponding EDS analysis shows that the main diffraction peaks of the alloy are occupied by Al (Figure 5b,d), with a few diffraction peaks of Mn and Mg, as well as weak diffraction peaks of Cr and Fe. Because Cu is used as the welded joint of the sample, the diffraction peak is detected, while the sample does not contain Cu.

**Figure 6:**
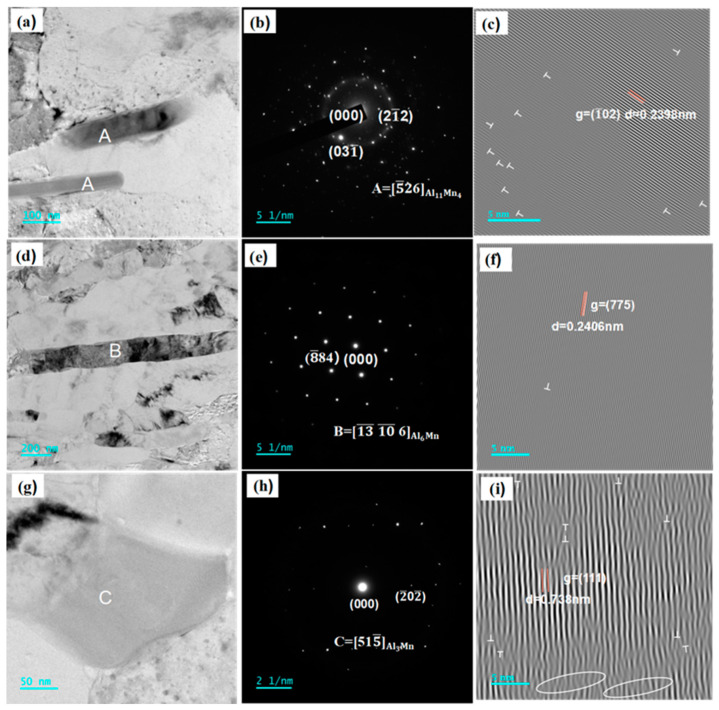
TEM, SAED, and IFFT of Al-4.6Mg alloy. (**a**,**d**,**g**) TEM bright field images of Al-4.6Mg alloy, (**b**,**e**,**h**) SAED images of Al-4.6Mg alloy, (**c**,**f**,**i**) IFFT images of Al-4.6Mg alloy.

Figure 6 shows the TEM morphologies, selected area electron diffraction (SAED) patterns, and inverse fast Fourier transformation (IFFT) images of the large precipitates of Al-4.6Mg alloy in the sensitized and desensitized states. The short rod-shaped precipitates were approximately 50 nm in diameter and 1 μm in length (Figure 6a), and, for phase identification, the SAED patterns of the precipitates were analytically calibrated (Figure 6b), and the crystal plane spacing in IFFT image was calculated (Figure 6c). The calibration result is consistent with the standard spectrum of the ν-Al_11_Mn_4_ phase, indicating that the precipitates are ν-Al_11_Mn_4_ phase structures. ν-Al_11_Mn_4_ is a triclinic system where the space group is P-1(2), and the cell parameters are as follows: a = 0.5052 nm, b = 0.8873 nm, c = 0.5034 nm, α = 89.7°, β = 99.8°, and γ = 104.9°. The theoretically calculated plane spacing of the phase in the (1$\overset{-}{0}$2) direction is 0.2378 nm (PDF # 47-1272), and the measured plane spacing is 0.2398 nm (Figure 6c). The measurement value is larger than the calculation value. During solution and rolling, the lattice is distorted and shows apparent dislocations (Figure 6c), which are marked by T. The ν-Al_11_Mn_4_ phase is formed by the following reactions [28]:

6Al + Mn → Al_6_Mn (1)

2Al_6_Mn + Mn → 3Al_4_Mn (2)

11Al_4_Mn + 5Mn → 4Al_11_Mn_4_ (3)

The specific formation process is as follows. The Al atoms diffuse rapidly to the local Mn matrix at the contact point between Al and Mn particles to form the Al_6_Mn phase, since the diffusion coefficient of Al is higher than that of Mn; the Al_6_Mn phase and local Mn atoms further form the Al_3_Mn phase, and the Al_3_Mn phase and the local excess Mn atoms form the ν-Al_11_Mn_4_ phase. The results show that the Al_6_Mn phase precipitates in the sensitization process, and the Al_6_Mn phase transforms into the ν-Al_11_Mn_4_ phase during desensitization. Tamara Radeti [29] found the ν-Al_11_Mn_4_ phase in the as-cast AA5083 aluminum alloy, and its heat treatment mode is largely consistent with that in the present paper.

The long rod-shaped precipitates were approximately 90 nm in diameter and 1.8 μm in length (Figure 6d), and their SAED pattern was analyzed and calibrated for phase identification (Figure 6e). The crystal plane spacing in the IFFT image was calculated (Figure 6f). The calibration result is consistent with the standard spectrum of the Al_6_Mn phase, indicating that these precipitates are Al_6_Mn phase structures. The space group of the Al_6_Mn phase is PE, and its cell size is a = b = c = 1.67 nm. The theoretically calculated interplanar spacing of the Al_6_Mn phase in the (775) direction is 0.2405 nm (PDF # 00-039-0590), and the measured interplanar spacing is 0.2406 nm (Figure 6f). The results show that the Al_6_Mn phase has less lattice distortion and a lower dislocation density during rolling, and the analysis results are consistent with the IFFT calibration results (Figure 6f). The Al_6_Mn phase is the most Al-rich intermetallic compound in the Al-Mn system [30,31]. When the content of the Mn element in Al-Mn alloy reaches a certain level, the solute content increases, which leads to the precipitation of the Al_6_Mn intermetallic compound. Many researchers have studied the orientation relationship between the Al_6_Mn phase and the matrix, as well as the effect of Mn addition on the dispersion behavior [32]. Al_6_Mn dendrites with different development degrees appear in the microstructure of Al-Mn alloy. In Al-4Mn alloy, there are a few bulk Al_6_Mn crystals in the center, whereas the bulk Al_6_Mn phase and the feather-like Al_6_Mn phase are seen in the Al-6Mn alloy [33]. To identify a precipitate with a side length of approximately 100 nm as a square phase structure (Figure 6g), the SAED pattern was analyzed and calibrated (Figure 6h). The high-resolution image was obtained by IFFT (Figure 6i). The calibration results are in agreement with the standard spectra of the Al_3_Mn phase, indicating that it is an orthorhombic structure and the space group is Pbnm. The cell size of the Al_3_Mn phase is a = 1.259 nm, b = 14.8 nm, and c = 11.42 nm. The structure of the Al_3_Mn phase has been extensively studied [34], but the exact space groups and atomic positions of Al_3_Mn intermetallic compounds remain controversial [35]. The actual crystal plane spacing of the phase in the (111) direction is 0.749 nm (PDF # 00-026-0028), and the theoretically calculated value is 0.738 nm. The theoretically calculated value is smaller than the measurement value, suggesting that apparent lattice distortion and dislocations occur in the Al_3_Mn phase during rolling. In the IFFT diagram (Figure 6i), dislocations and aberrations are marked by Ts and circles, respectively.

**Figure 7:**
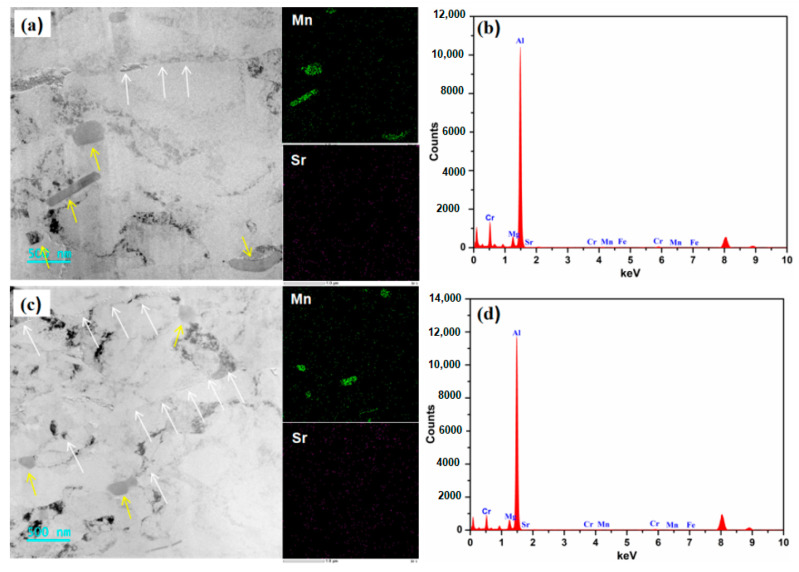
TEM-EDS image of Al-4.6Mg-0.1Sr alloy. (**a**,**b**) TEM and Mapping images of Al-4.6Mg-0.1Sr alloy, (**c**,**d**) EDS images of Al-4.6Mg-0.1Sr alloy.

Figure 7 shows the TEM-EDS analysis results of the Al-4.6Mg-0.1Sr alloy. The precipitates of Al-4.6Mg-0.1Sr alloy are mainly composed of a bar, block, and sphere. The grain boundaries and phase boundaries (marked by white arrows) can be seen from the TEM bright-field phase contrast images of Al-4.6Mg-0.1Sr alloy. The TEM-EDS image (Figure 7a,c) shows that Mn-rich phases are in the majority; no enrichment of the Mg-rich phase was found in the element distribution diagram, indicating that Sr inhibited the precipitation of the Mg-rich phase. Some Mn-rich phase particles are marked by yellow arrows. The morphology and size distribution of the Mn-rich phase are quite different from those of other phases, which indicates its unique features in terms of nucleation, growth, and element content. It can be seen from the corresponding EDS analysis diagram (Figure 7b,d) that the main diffraction peaks of the alloy are occupied by Al, and there are a few diffraction peaks of Mn, Cr, and Mg, as well as weak diffraction peaks of Sr and Fe. The diffraction peak of Cr is obvious. No enrichment of the Cr-rich phase can be found in the distribution diagram of Cr, indicating that part of the Cr was solubilized in the α-Al matrix, which played necessary roles supplementary role in solid solution strengthening, and a large amount of Cr formed the Al_6_(Mn Cr) phase. Due to the limited scanning area of TEM, larger Al_6_(Mn Cr) phase particles could not be detected. Sr mainly formed a rod-like Al_4_Sr phase and high-Mg ternary τ-Al_38_Mg_58_Sr_4_ phase, but they could not be detected because of the limitations of TEM scanning.

**Figure 8:**
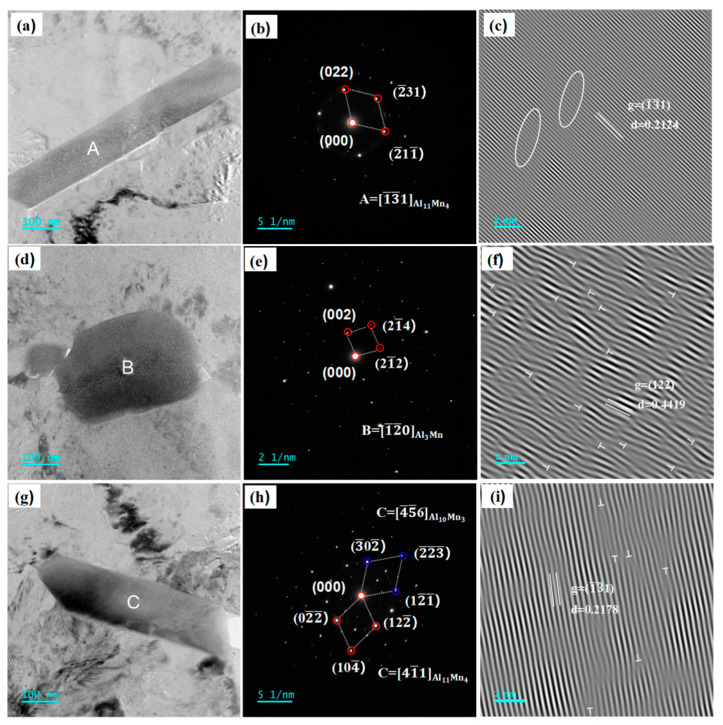
TEM, SAED, and IFFT of Al-4.6Mg-0.1Sr alloy. (**a**,**d**,**g**) TEM bright field images of Al-4.6Mg-0.1Sr alloy, (**b**,**e**,**h**) SAED images of Al-4.6Mg-0.1Sr alloy, (**c**,**f**,**i**) IFFT images of Al-4.6Mg-0.1Sr alloy.

The nano-precipitates of the Al-4.6Mg-0.1Sr alloy in sensitization–desensitization heat treatment were analyzed by TEM, SAED, and IFFT to investigate the effect of Sr on their morphology and distribution. As seen from the TEM bright-field image (Figure 8a), the short rod-shaped nano-precipitates had a diameter of approximately 80 nm and a length of approximately 1 μm. Their SAED pattern was analyzed and calibrated (Figure 8b), and the crystal plane spacing was calculated based on the IFFT image (Figure 8c). The calibration result is consistent with the standard diagram of the ν-Al_11_Mn_4_ phase, indicating that the precipitates are ν-Al_11_Mn_4_ phase structures. The calculated plane spacing of the ν-Al_11_Mn_4_ phase in the (1$\overset{-}{3}\overset{-}{1}$) direction is 0.2114 nm (PDF # 47-1272), and the measured plane spacing is 0.2124 nm (Figure 8c), which is slightly larger. The difference is due to the lattice distortion during rolling, which is marked by circles (Figure 8c).

As seen from the TEM bright-field image (Figure 8d), the square-shaped nano-precipitates had a width of about 130 nm and a length of about 200 nm. Their SAED pattern was analyzed and calibrated (Figure 8e), and the crystal plane spacing in the IFFT image was calculated (Figure 8f). Based on the results, the precipitates are identified as Al_3_Mn phase structures. The actual crystal plane spacing of the Al_3_Mn phase in the (122) direction is 0.444 nm, and the calculated IFFT plane spacing is 0.4419 nm, which is slightly smaller. The discrepancy is mainly due to the lattice distortion of the Al_3_Mn phase caused by rolling deformation and frequent dislocations. The dislocations are marked by Ts (Figure 8f).

The short rod-shaped precipitates were approximately 80 nm in diameter and 1 μm in length (Figure 8g). Their SAED pattern was analyzed, and two distinct sets of diffraction spots were found (Figure 8h). The strong diffraction spots and the weak diffraction spots were calibrated. The results were consistent with the standard spectra of ν-Al_11_Mn_4_ and Al_10_Mn_3_, respectively, suggesting that the ν-Al_11_Mn_4_ phase and Al_10_Mn_3_ phase dominated. The Al_10_Mn_3_ phase belongs to a cubic system. Its space group is P63, and the cell parameters are as follows: a = b = 0.751 nm, c = 0.7779 nm, α = β = 90°, and γ = 120°. The high-resolution image was obtained by IFFT (Figure 8i), and the crystal plane spacing was calculated to be 0.2178 nm. The actual crystal plane spacing of ν-Al_11_Mn_4_ in the (1$\overset{-}{3}\overset{-}{1}$) direction was 0.2114 nm, smaller than the calculation value because of the lattice distortion caused by rolling deformation (Figure 8i). The addition of 0.1% Sr to Al-4.6Mg alloy markedly changed the morphology of the nano-precipitates; long rod-shaped precipitates and Mg-rich β-Al_3_Mg_2_ precipitates decreased while square precipitates increased.

### 3.4. Analysis of Mechanical Properties

The mechanical properties of the two alloys after sensitization–desensitization heat treatment are shown in Table 3.

The tensile strength and yield strength of Al-4.6Mg alloy were 372.72 MPa and 288.28 MPa, respectively, and the elongation at break was 22.42%. The Sr addition contributed to the improvement of their mechanical properties. The tensile strength and yield strength of Al-4.6Mg-0.1Sr alloy increased to 378.70 MPa and 327.53 MPa, respectively, and the plasticity decreased (Table 3). The significant enhancement in the tensile strength and yield strength and the decrease in the plasticity of the Sr-added alloy are attributed to the increase in rolled grains and the refinement of alloy grains resulting from the inhibition of dynamic recovery and dynamic recrystallization by Sr.

**Figure 9:**
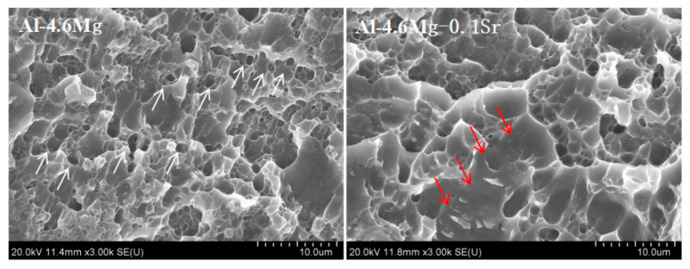
Fracture morphology of the two alloys.

In order to further study the effect of Sr on the mechanical properties of Al-4.6Mg alloy, the fracture morphology of the tensile specimen was observed and analyzed by SEM (Figure 9). Al-4.6Mg alloy has a large number of equiaxed dimples at its ports, and the equiaxed dimples have a high density of distribution (indicated by white arrows), which results in the high ductility of the alloy. By combining SEM and EDS analysis, it was found that the content of Mg in the edges of equiaxed dimples was high, there was no Mn or Cr, and the β-Al_3_Mg_2_ phase appeared at the fracture site, suggesting that the fracture occurred at the Mg-rich phase. The α-Al matrix bears most of the plastic deformation during fracture because of the difference in plasticity between the α-Al matrix and β-Al_3_Mg_2_ phase, and elliptical dimples are formed around the β-Al_3_Mg_2_ phase due to the distribution of the β-Al_3_Mg_2_ phase. The shape of the dimples is irregular, and their distribution is not uniform. With the addition of Sr, the equiaxed dimple density of the Al-4.6Mg-0.1Sr alloy fracture became lower, and a quasi-cleavage fracture appeared (indicated by red arrows). The results show that, during the tensile process, the fracture of the Al-4.6Mg-0.1Sr alloy is ductile, but with a share of brittle fracture, leading to a plasticity decrease. It was also found that there were Mg and Sr elements in the edges of the dimples, indicating that the fracture occurred at the β-Al_3_Mg_2_ phase and Al_4_Sr phase. Compared with the α-Al matrix, the Al_4_Sr phase is more rigid and brittle. It firstly breaks and forms a quasi-cleavage fracture after stress concentration during the tensile process; then, the cracks extend into the Al matrix and tear it to form equiaxed dimples, and, finally, material failure occurs.

### 3.5. Analysis of Enhancement Mechanism

The mechanical properties of Al-4.6Mg alloy are improved mainly by work hardening and solution strengthening. It is shown in Table 3 that the addition of 0.1 wt.% Sr can improve the mechanical properties of Al-4.6Mg alloy through fine grain strengthening, τ-Al_38_Mg_58_Sr_4_ dispersion strengthening, and precipitation strengthening. According to polycrystalline plastic deformation theory, due to the high energy at the grain boundary, more energy is needed for the dislocation slip across the grain boundary than for the intracrystalline slip. The decrease in grain size can contribute to the alloy’s strength enhancement through dislocation slip inhibition. The strength enhancement by Sr addition through grain refinement can be represented by the Hall–Petch relation [36].

***σ_gb_* = *σ*_0_ + *k_HD_d*^−1/2^** (4)

where ***σ_gb_*** is the strength increase induced by the decrease in the grain size of the alloy; *σ*_0_ is the frictional stress required to prevent dislocation slip; ***k*** is the coefficient of the effect of the orientation difference between adjacent grains on dislocation movement, which is commonly known as grain boundary resistance; ***k_HD_*** is the Hall–Petch coefficient and is about 0.14 MPa m^1/2^ [37] for Al-Mg alloy; ***d*** is the average grain size of polycrystals. According to this equation, the strengthening effect is inversely proportional to the grain size of the alloy. In other words, with the decrease in the grain size, the fraction of grain boundary per unit volume increases, which indicates that the mechanical properties of the alloy are enhanced. The average grain size of Al-4.6Mg alloy is 6.86 μm, while that of Al-4.6Mg-0.1Sr alloy is 5.39 μm after sensitization–desensitization heat treatment. According to Equation (4), after fine grain strengthening by Sr addition, the strength enhancement is about 6.85 MPa. The strength enhancement is limited because the influence of grain refinement after adding Sr is slight.

The main reason for Sr-induced dispersion strengthening is that a large number of fine, dispersed, high-Mg ternary phases τ-Al_38_Mg_58_Sr_4_ can be formed in the alloy with Sr addition, and the τ-Al_38_Mg_58_Sr_4_ phase can contribute to dispersion strengthening and improve the yield strength of the alloy, but the increase in the size of the Al_4_Sr phase cannot induce effective dispersion strengthening.

The dispersion strengthening mechanism of the second-phase particle τ-Al_38_Mg_58_Sr_4_ includes dislocation cut-through and dislocation bypass. The results show that the Orowan dislocation-bypassing mechanism dominates when the particle diameter is larger than 5 nm. Therefore, the contribution of the τ-Al_38_Mg_58_Sr_4_ particles to the yield strength enhancement of the alloy can be expressed by Equation (5) [38].

(5) $$∆\sigma =M\frac{0.4Gb}{\pi \sqrt{1-v}}\frac{ln(\frac{2\overset{-}{r}}{b})}{\lambda }$$

where

(6) $$\lambda =2\overset{-}{r}(\sqrt{\frac{\pi }{4f}}-1)$$

**∆*σ*** is the strength increment, which is proportional to the volume fraction of the precipitates and inversely proportional to the size of the precipitates. The Poisson’s ratio *v* = 0.34 [39]. R is the average radius of the spherical precipitates, and the cube structure $\overset{-}{r}$ = $\sqrt{2}/3r$. The spherical precipitate of the Al-4.6Mg-0.1Sr alloy is a τ-Al_38_Mg_58_Sr_4_ structure. The τ-Al_38_Mg_58_Sr_4_ phase particles were 60 nm in diameter, and the volume fraction of the τ-Al_38_Mg_58_Sr_4_ phase was calculated by the Image-Pro Plus (IPP) software, which was about 2.31 × 10^−3^m^−3^. The related parameters were substituted into Equations (5) and (6). It is estimated that the strength of the spherical τ-Al_38_Mg_58_Sr_4_ phase precipitate is about 8.203 MPa.

The solution strength enhancement of Al-4.6Mg alloy is induced by solute atoms in the matrix metal, which results in lattice distortion to a certain extent. The lattice distortion increases the resistance against dislocation movement and makes the slip difficult. Therefore, the strength and hardness of the solid solution alloy are increased. The solute atoms directly enhance the Al matrix by preventing dislocations, and they form solute clusters by diffusing to the dislocated nuclei and dragging them. The strength increments under these two modes of interaction from the beginning can be estimated by Equation (7) [40]:

∆σ_ss_ = KC^n^ (7)

where K = 12.1 MPa/wt.%; ***n*** is the relevant material constant and ***n*** = 1; C is the concentration of substitutional Mg solutes in the Al crystal lattice [41]. According to the literature method [37], the C values of Al-4.6Mg alloy and Al-4.6Mg-0.1Sr alloy are 2.86 at.% and 3.36 at.%, respectively. By calculation using Equation (7), it is found that the increment (∆σ_ss_) induced by the Sr element is 4.85 MPa.

The strength and hardness of the alloy increase after rolling due to the grain boundary sliding and dislocation entanglement, which leads to grain elongation, breakage, and fiber formation. In addition, the residual stress in the material decreases its plasticity and toughness. The dislocation structure in the rolled material can effectively suppress the dislocation slip, and the increased strength is calculated by Taylor’s equation, as shown in Formula (8).

(8) $$∆\sigma_{DS}MGb\sqrt{\rho }$$

For Al-Mg alloy, Taylor factor M = 3.06, shear modulus ***G*** = 27 GPa, material constant α = 0.2, Burgers vector ***b*** = 0.286 nm [42], and ***ρ*** is the geometric dislocation density (GND) of the alloy. The GND of Al-4.6Mg alloy is 8.903 × 1013 m^−2^, and that of Al-4.6Mg-0.1Sr alloy is 1.325 × 1014 m^−2^. The yield strength of Al-4.6Mg-0.1Sr alloy calculated by Equation (4) increases by about 9.807 MPa, and the dislocation density also increases. The addition of 0.1 wt.% Sr to Al-4.6Mg alloy results in an increase in yield strength of ***σ_gb_*** + **∆*σ*** +**∆*σ_ss_*** + **∆*σ_DS_*** = 29.71 MPa, while the experimental value is 29.25 MPa.

## 4. Conclusions

The effects of Sr on the microstructure of Al-4.6Mg alloy and the strengthening mechanisms were studied in this paper. The following conclusions were reached.

(1) The main precipitation phases of Al-4.6Mg alloy were β-Al_3_Mg_2_, Al_6_Mn, and Al_6_(Mn Cr), and nano-precipitates Al_3_Mn and Al_11_Mn_4_ were found. The Sr element effectively inhibited the precipitation of the β-Al_3_Mg_2_ phase, and the number and size of the β-Al_3_Mg_2_ phase decreased, resulting in a rod-like Al_4_Sr phase and spherical τ-Al_38_Mg_58_Sr_4_ precipitation phase.
(2) The addition of Sr in Al-4.6Mg alloy resulted in increased LABs and GND and decreased HABs and grain sizes. The addition of Sr decreased the content of recrystallized grains and subgrains and increased the content of deformed grains. Sr increased the recrystallization temperature, hindered the recrystallization process, and restrained the growth of recrystallized grains under sensitization–desensitization heat treatment.
(3) The mechanical properties of Al-4.6Mg alloy were enhanced by adding Sr, and its yield strength was improved by fine grain strengthening, dispersion strengthening, solution strengthening, and work hardening. The experimentally determined yield strength increments showed good agreement with the values determined by the theoretical analysis.
(4) This study provides theoretical guidance for the application of Sr elements in Al-4.6Mg alloys and the development of Al-Mg-Sr alloys.

## Figures and Tables

**Table 1:** Chemical composition of the Al-4.6Mg-0.1Sr alloy (wt.%).

Alloy	Mg	Sr	Ti	Mn	Cr	Al
Al-4.6Mg-0.1Sr	4.53	0.09	0.02	0.57	0.15	Bal

**Table 2:** Second-phase component content of the two alloys (wt.%).

Alloy	Location	Mg	Al	Cr	Mn	Sr
Al-4.6Mg	a	3.2	81.3	1.7	13.6	/
	b	0.9	73.9	2.4	22.4	/
	c	2.5	79.8	1.6	15.6	/
	d	4.7	93.7	0.3	0.6	/
Al-4.6Mg- 0.1Sr	e	1.1	80.9	0.7	15.7	
	f	5.2	78.2	0.1	0.2	13.3
	g	2.2	82.1	1.1	13.5	/
	h	9.8	71.4	2.5	11.4	4.4
	i	5.1	93.1	0.1	0.6	/

**Table 3:** Mechanical properties of the two alloys.

Alloy	Tensile Strength/MPa	Yield Strength/MPa	Elongation at Break/%
Al-4.6Mg	372.72	298.28	22.42
Al-4.6Mg-0.1Sr	378.70	327.53	18.04

## Data Availability

All data that support the findings of this study are included within the article.
